# Stattic Enhances Radiosensitivity and Reduces Radio-Induced Migration and Invasion in HCC Cell Lines through an Apoptosis Pathway

**DOI:** 10.1155/2017/1832494

**Published:** 2017-10-26

**Authors:** Gang Xu, Lihua Zhu, Yan Wang, Yawei Shi, Aihua Gong, Chaoyang Wu

**Affiliations:** ^1^Department of Radiation Oncology, Affiliated People's Hospital, Jiangsu University, Zhenjiang, Jiangsu 212002, China; ^2^School of Medicine, Jiangsu University, Zhenjiang, Jiangsu 212013, China

## Abstract

**Purpose:**

Signal transducer and activator of transcription factor 3 (STAT3) is involved in tumorigenesis, development, and radioresistance of many solid tumors. The aim of this study is to investigate the effects of stattic (an inhibitor of STAT3) on the radiosensitivity and radio-induced migration and invasion ability in hepatocellular carcinoma (HCC) cell lines.

**Methods:**

HCC cells were treated with stattic, and cell survival rate was analyzed through CCK-8 assay. Radiosensitivity was evaluated using cloning formation analysis; STAT3, p-STAT3, and apoptosis related proteins were detected by western blot. Radio-induced migration and invasion ability in HCC cells were analyzed by wound-healing assay and transwell test.

**Results:**

Stattic inhibits the expression of p-STAT3 and reduces cell survival in a dose-dependent manner in HCC cell lines, and the IC50 values for Hep G2, Bel-7402, and SMMC-7721 are 2.94 *μ*M, 2.5 *μ*M, and 5.1 *μ*M, respectively. Cloning formation analysis shows that stattic enhances the radiosensitivity of HCC cells. Wound-healing assay and transwell test show that stattic inhibits radio-induced migration and invasion. Further study indicates that stattic promotes radio-induce apoptosis through regulating the expression of apoptosis related proteins in HCC cells.

**Conclusion:**

Stattic enhances radiosensitivity and reduces radio-induced migration and invasion ability in HCC cells probably through apoptosis pathway.

## 1. Introduction

Hepatocellular carcinoma (HCC) is a popular carcinoma and is the second leading cause of cancer-related death currently [1]. Surgery is the most effective therapy for HCC, but only approximately 10–30% of cases are operable, and the tumor always recurs and metastasizes to other organs after surgery. The overall 5-year survival rate is only 5–10% [2–4]. Radiotherapy is an effective treatment for locally advanced HCC, but its clinical application is limited because of the poor radiation tolerance of the liver and its surrounding tissues [5, 6]. As new radiotherapy technology has been used, such as intensity modulated radiotherapy (IMRT), image guided radiotherapy (IGRT), and three-dimensional conformal radiotherapy (3D-CRT), radiation-induced liver injuries have moderately decreased, but the technical improvement is limited. Therefore, development of an effective radiosensitizer to enhance the radiosensitivity of hepatoma cells will play an important role in the treatment of HCC.

Signal transducer and activator of transcription factor 3 (STAT3) is cytoplasmic, that upregulates the expression of genes related to cancer proliferation, survival, and invasion, and is involved in the tumorigenesis, development, migration, and invasion of many solid tumors [7, 8]. STAT3 plays an important role in the differentiation and tumorigenesis of hepatocellular carcinoma [9–11]. The phosphorylated protein level of STAT3 was higher in HCC tissues than that in the normal liver and surrounding tissues [12]. Targeting STAT3 seems to be a novel approach to prevent and treat HCC.

Many studies have shown that blocking the activation of STAT3 will reduce the survival rate, proliferation, migration, and invasion of cancer cells. A recent study showed that there were three methods to target STAT3, including modulation of upstream regulators, RNA interference, and targeting STAT3 protein directly [13]. Modulation of upstream regulators may not block STAT3 completely due to the cross-talk between many molecular pathways, and RNA interference is still a long way from being approved for clinical usage. Therefore, small molecular inhibitor targeting STAT3 might be a better method to inhibit STAT3. Stattic is such an inhibitor targeting the SH2- domain of STAT3 [14]. Some researchers found that stattic could enhance the radiotherapy and/or chemotherapy sensitivity of head and neck squamous cell carcinoma, nasopharyngeal carcinoma, colorectal carcinoma, and esophageal cancer [15–18]. Whether stattic could enhance the radiosensitivity of HCC cells has not been reported yet. In this study, we investigated the effects of stattic on cell survival, migration, invasion, and radiosensitization in HCC cell lines.

## 2. Materials and Methods

### 2.1. Cell Culture

Hep G2, Bel-7402, and SMMC-7721 cells were obtained from the School of Medical Sciences and Laboratory Medicine, Jiangsu University, and cultured in Dulbecco's modified Eagle's medium (DMEM) (HyClone, USA) with 10% fetal bovine serum (HyClone, USA), 100 *μ*g/ml penicillin, and 0.25 *μ*g/ml streptomycin at 37°C and 5% CO_2_.

### 2.2. CCK-8 Assay

Hep G2, Bel-7402, and SMMC-7721 cells (3 × 10^3^ cells/well) were seeded in 96-well plates for 24 h and then were treated with different doses of stattic (0–32 *μ*M). Cell counting kit-8 (CCK-8) (Vazyme Biotech, USA) was used for cell survival analysis. In brief, 10 *μ*l CCK-8 solution was added to the cells and incubated for 1 h at 37°C. The absorbance at 450 nm was measured by a microplate reader (BD, USA).

### 2.3. Wound-Healing Assay

Hep G2, Bel-7402, and SMMC-7721 cells were seeded (2 × 10^6^/well) in a 6-well plate. A wound was made by scratching a confluent monolayer with the tip of a 10 *μ*l pipette. Nonadherent cells were washed off with sterile PBS. HCC cells were treated with stattic (Sigma, USA) or the same volume of DMSO (Sigma, USA), irradiated with 4 Gy of X-ray, and then placed in the incubator to culture for 24 h. Pictures were taken by inverted microscope (Nikon Ti, Japan) at 100x magnifications. The width of the scratches was measured using Motic Image Plus 2.2S software (Shimadzu, Japan).

### 2.4. Transwell Invasion Assay

Hep G2, Bel-7402, and SMMC-7721 cells (2 × 10^6^/well) were seeded in serum-free medium on transwell inserts (6.5 mm, 8 *μ*m pores, Corning, USA) coated with 1 mg/ml matrigel (Becton Dickinson, USA). HCC cells were treated with stattic (Sigma, USA) or the same volume of DMSO (Sigma, USA), irradiated with 4 Gy of X-ray, and then placed in the incubator to culture for 24 h. Cells from the upper side of the insert were scraped away, and then the inserts were fixed and stained. Invaded cells were counted under an inverted microscope.

### 2.5. Colony Formation Assay

Hep G2, Bel-7402, and SMMC-7721 cells were seeded in 6-well plates at different densities (200, 200, 400, 400, and 800 cells for 0 Gy, 2 Gy, 4 Gy, 6 Gy, and 8 Gy groups separately), and, 24 h later, the cells were treated with stattic or DMSO for 4 h and irradiated with 0 to 8 Gy of X-ray using a linear accelerator (Siemens, GER). After the irradiation, the cells were cultured at 37°C for 14 days, fixed with methanol, and stained with 0.05% crystal violet (Sigma, USA), and the number of colonies consisting of 50 or more cells was counted, and the surviving fraction was calculated. The survival curves were plotted with a single-hit multitarget model using GraphPad 5 (San Diego, CA), and the values of *D*0, *Dq*, and SER were calculated.

### 2.6. Western Blotting Analysis

The proteins were separated by 10% sodium dodecyl sulfate-polyacrylamide gel electrophoresis (SDS-PAGE) (Beyoutime, China) and then transferred to polyvinylidene fluoride membranes (Millipore, USA), blocked for 2 h with 5% nonfat milk. The membranes were incubated with primary antibodies against p-STAT3 (1 : 2000, Cell Signaling Technology, USA), STAT3 (1 : 1000, Cell Signaling Technology, USA), Bcl-2 (1 : 1000, Cell Signaling Technology, USA), Bax (1 : 1000, Cell Signaling Technology, USA), and *β*-actin (1 : 5000, Cell Signaling Technology, USA) overnight at 4°C. Next, the membranes were incubated with horseradish peroxidase- (HRP-) conjugated secondary antibodies for 1 h at room temperature. The blots were visualized using the Super Signal West Femto kit (Pierce, USA). Activity of STAT3 = p-STAT3/STAT3. Image J software (NIH, USA) was used for semiquantitation analysis on western blotting images, and the value of control was taken as 100 in each cell line.

### 2.7. Statistical Analysis

All statistical analysis was performed using SPSS 19.0 software (SPSS, USA). Data were represented as mean ± standard deviation; all experiments were performed in triplicate. Student's *t*-test or one-way analysis of variance (ANOVA) was used for statistical analysis. In ANOVA, when a significant difference was apparent, Dunnett test was used in multiple comparisons of means.

## 3. Results

### 3.1. Stattic Inhibits the Survival Rate of HCC Cells

**Figure 1:**
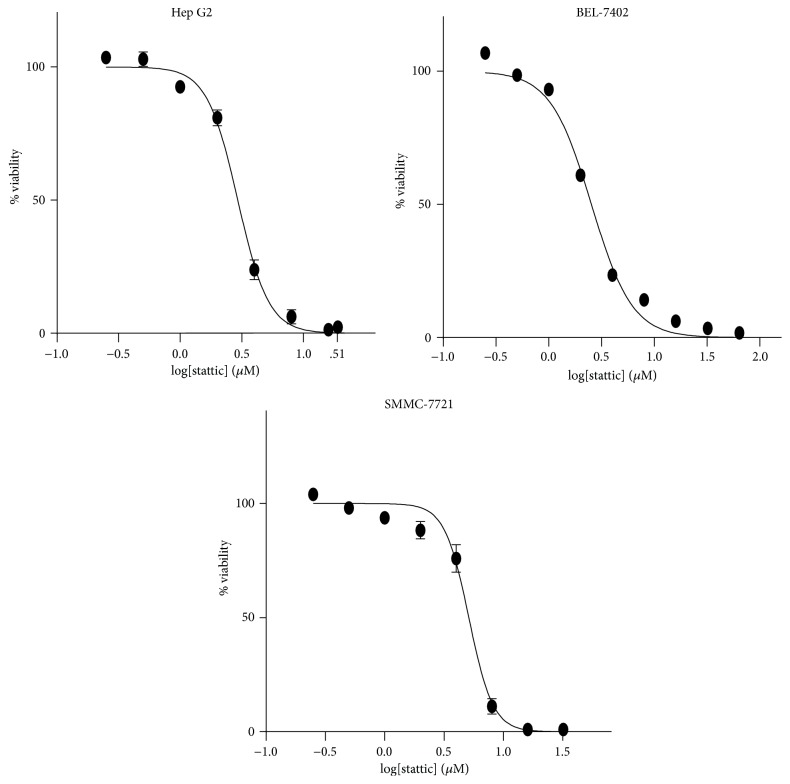
Stattic inhibits cell survival of HCC cell lines. Hep G2, Bel-7402, and SMMC-7721 cells were treated with stattic (0–32 *μ*M) for 48 h before the cell viability was measured with CCK-8 kit. IC50 values were calculated from these curves using GraphPad Prism 5 software. Each data point is the mean of three independent experiments ± SD.

We exposed three HCC cell lines to different concentrations of stattic (0–32 *μ*M) for 48 h. As shown in Figure 1, CCK-8 assay showed that stattic inhibited the viability of Hep G2, Bel-7402, and SMMC-7721 cells in a dose-dependent manner. The IC50 values for Hep G2, Bel-7402, and SMMC-7721 were 2.94 *μ*M, 2.5 *μ*M, and 5.1 *μ*M, respectively.

### 3.2. Stattic Inhibits Radio-Induced STAT3 Activation in HCC Cells

**Figure 2:**
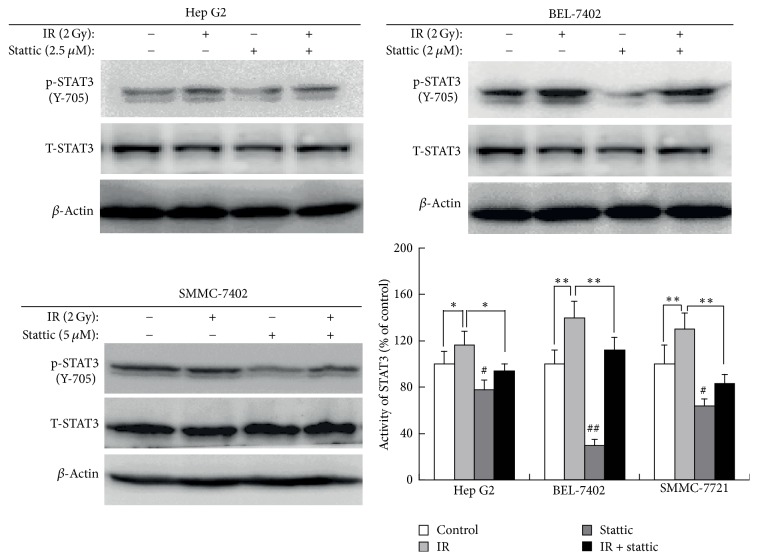
Stattic inhibits radio-induced STAT3 activation in HCC cell lines. Hep G2, Bel-7402, and SMMC-7721 cells were pretreated with stattic or DMSO for 24 h and then were irradiated with 2 Gy of X-ray using a linear accelerator. Expression of p-STAT3 and t-STAT3 was examined by western blot. The activity of STAT3 = p-STAT3/t-STAT3. Each experiment was performed three times. ^#^*p* < 0.05, ^##^*p* < 0.01 versus control group. ^*∗*^*p* < 0.05, ^*∗∗*^*p* < 0.01 versus irradiation group. Data were expressed as mean ± SD.

To investigate the effect of stattic on radiation-induced STAT3 activation in HCC cells, we exposed three HCC cell lines pretreated with stattic or DMSO to radiation (2 Gy) and then examined the protein levels of STAT3 and p-STAT3 by western blot analysis. As shown in Figure 2, radiation upregulated the expression of p-STAT3; however, the expression of p-STAT3 was decreased significantly in the cells treated with stattic. Stattic inhibited radio-induced STAT3 activation in HCC cell lines.

### 3.3. Stattic Inhibits Radio-Induced Migration and Invasion Ability in HCC Cells

**Figure 3:**
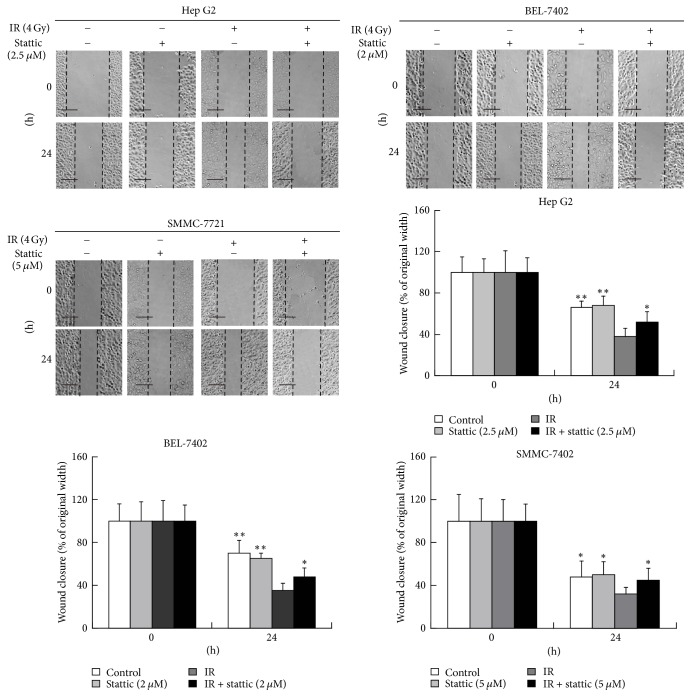
Stattic inhibits radio-induced migration in HCC cell lines. A wound was made by scratching a confluent monolayer with the tip of a 10 *μ*l pipette. HCC cells pretreated with stattic or DMSO were irradiated with 4 Gy of X-ray and then placed in the incubator to culture for 24 h. Pictures were taken by an inverted microscope (100 magnifications). The width of the scratch was calculated using Motic Image Plus 2.2S. Each experiment was performed three times. ^*∗*^*p* < 0.05, ^*∗∗*^*p* < 0.01 versus irradiation group; data were expressed as mean ± SD. Scale bar = 100 *μ*m.

**Figure 4:**
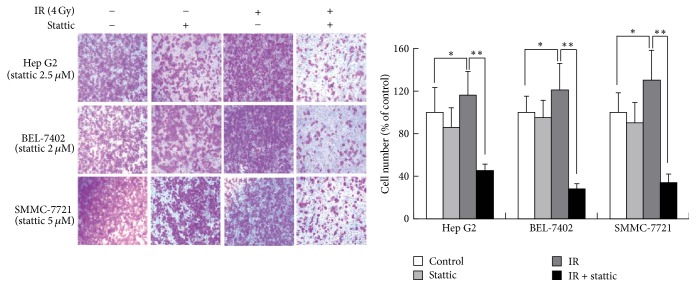
Stattic inhibits radio-induced invasion in HCC cell lines. HCC cells were seeded in serum-free medium on transwell inserts (6.5 mm, 8 *μ*m pores) coated with matrigel, and treated with stattic or DMSO for 24 h, irradiated with 4 Gy of X-ray, and then placed in the incubator to culture for 24 h. The inserts were fixed and stained with 0.05% crystal violet. Invaded cells were counted under an inverted microscope (100 magnifications). Each experiment was performed three times. ^*∗*^*p* < 0.05, ^*∗∗*^*p* < 0.01, versus irradiation group. Data were expressed as mean ± SD.

We analyzed the migration and invasion ability of HCC cells using a wound-healing assay and a transwell test. The mean width of the wound was decreased in the radiation group (4 Gy) compared to that of the control and was significantly increased in the radiation combined with stattic group (Figure 3). The results of the transwell test demonstrated that radiation significantly enhanced invasion in HCC cells and that stattic inhibited this effect of radiation. These results showed that stattic could inhibit radio-induced invasion and migration in HCC cells (Figure 4).

### 3.4. Stattic Enhances the Radiosensitivity of HCC Cells

**Figure 5:**
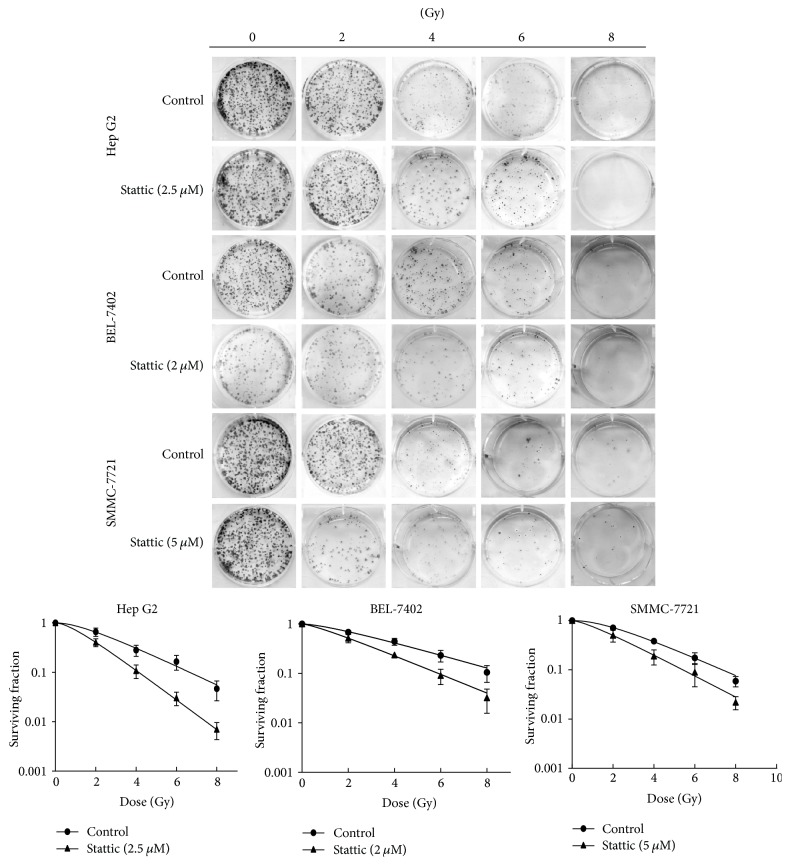
Stattic enhances radiosensitivity in HCC cell lines. HCC cells were plated in 6-well plates, treated with stattic or DMSO for 4 h, and then irradiated with 0 to 8 Gy of X-ray using a linear accelerator. The cells were grown at 37°C for 14 days, and the number of colonies consisting of 50 or more cells was counted. Each experiment was performed at least three times. The dose-survival curves were plotted and the values of *D*0, *Dq*, and SER were calculated using GraphPad 5 software.

Colony formation assays with radiation (0–8 Gy) showed that radiation caused a dose-dependent cytotoxic effect on HCC cells. Pretreatment with stattic sensitized Hep G2, Bel-7402, and SMMC-7721 cells and successfully enhanced the effects of radiation (Figure 5). The radiosensitization effects of stattic in HCC cells are summarized in Table 1.

### 3.5. Stattic Promotes Radio-Induced Apoptosis in HCC Cells

**Figure 6:**
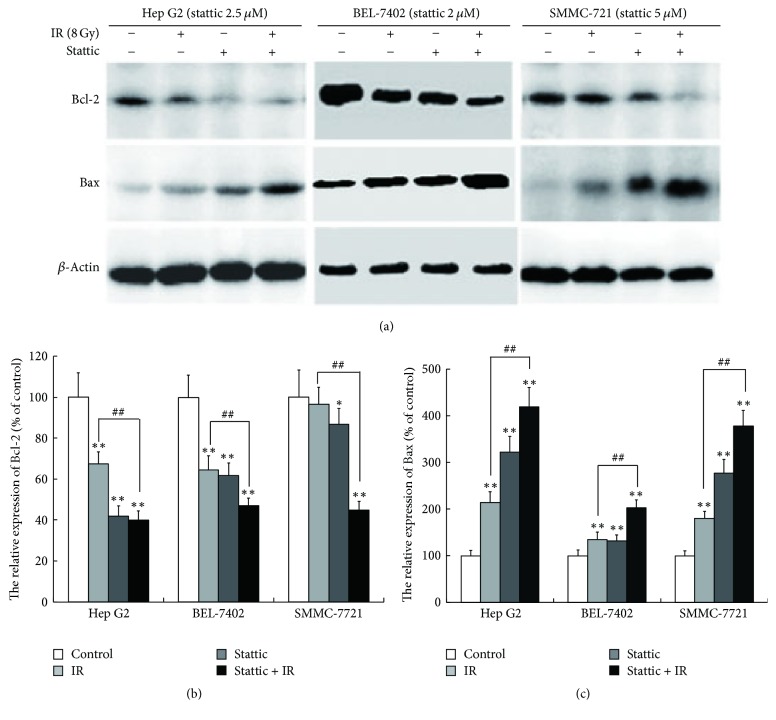
Stattic promotes radio-induced apoptosis in HCC cell lines. HCC cells were plated in 6-well plates, treated with stattic or DMSO for 4 h and irradiated with 8 Gy using a linear accelerator, and then placed in the incubator to culture for 24 h. The expression of Bcl-2 and Bax was examined by western bolt analysis. (a) The western blot images of Bcl-2 and Bax expression; (b) the relative expression of Bcl-2, ^*∗*^*p* < 0.05; (c) the relative expression of Bax. The expression of Bcl-2 and Bax in control group was taken as 100. ^*∗∗*^*p* < 0.01 versus control group, ^##^*p* < 0.01 versus irradiation group. Each experiment was performed at least three times.

We measured the expression of apoptosis related proteins for the possible mechanism of stattic on the apoptosis. As shown in Figure 6, the expression of Bcl-2 was downregulated in the stattic and radiation (8 Gy) group. By contrast, the expression of Bax was upregulated compared to that in the normal control group. However, these effects became more pronounced in the stattic plus radiation group. These results indicate that stattic can promote radio-induced apoptosis.

## 4. Discussion

In our study, we found that stattic, an inhibitor of STAT3, inhibited the activation of STAT3 and cell survival in HCC cell lines in a dose-dependent manner. According to the IC50 of HCC cells and the preliminary experimental results of STAT3 phosphorylation assay, we determined the concentrations of stattic in the subsequent studies for different cell lines, and the dose of X-ray in different experiment was determined according to the results of pretest, such as 2 Gy in STAT3 phosphorylation assay, 4 Gy in wound-healing and transwell assay, and 8 Gy in apoptosis analysis.

Recently, ionizing radiation has been reported to promote migration and invasion of surviving cells in several cancers [19, 20]. STAT3 also contributes to migration in cancer cells, such as breast cancer, ovarian cancer, lung cancer, and gastric cancer [21–25], and inhibition of STAT3 would decrease the migration and invasion ability. In our study, we found that radiation enhanced the expression of p-STAT3, so we hypothesized that radiation promoted migration and invasion of HCC cells through enhancing activation of STAT3. The results showed that radiation with 4 Gy promoted the migration and invasion ability of HCC cells and stattic blocked the effect of radiation. Consistent with this finding, Hsu et al. also found that radiation promoted the invasion of lung cancer cells by STAT3-induced accumulation of Bcl-xL [24].

Recent studies showed that the STAT3 pathway mediated radioresistance in many malignant tumors. Kim et al. proved that the continued activation of STAT3 could lead to radioresistance in breast cancer cells [26]. There are also some other similar reports about the role of STAT3 in the radioresistance of A431 squamous cell carcinoma, glioma, and head and neck carcinoma [27–29]. Therefore, we supposed that inhibition the activation of STAT3 might enhance the radiosensitivity of HCC cells. In our study, X-ray irradiation activated STAT3, while the STAT3 inhibitor stattic decreased the expression of p-STAT3 protein in the radiation group. Cloning formation assay showed that stattic could increase the radiosensitivity of HCC cells, as shown in Table 1, the value of *D*0, *Dq*, and SF2 was decreased in stattic group, and the SER of *D*0 is 1.56, 1.60, and 1.69 in Hep G2, BEL-7402, and SMMC7721, respectively, which indicates that STAT3 may be a new target for cancer radioresistance. However, the mechanism is not yet clear. Therefore, we carried out further investigation into the mechanism.

The families of Bcl-2 protein and Bax protein were found to be the most important apoptosis protein. Among the families of apoptosis related protein, Bcl-2 and Bax were considered to occupy the leading position. These apoptosis associated proteins are not only related to the development of malignant tumors, but also involved in the radiation-induced apoptosis [30]. The results of our study also showed that stattic increased the expression of Bax and decreased the expression of Bcl-2, which suggested that inhibition of the STAT3 pathway could induce apoptosis of HCC cells. On the other hand, many studies showed that JAK/STAT3 pathway played an important role in apoptosis [31, 32]; whether JAK is involved in the radiation and stattic induced apoptosis is not clear yet, which needs to be confirmed in future study. Lu et al. reported that inhibiting the STAT3 pathway by FTY720 promoted the apoptosis of cholangio carcinoma cells [33], which was consistent with our results. Interestingly, we found that apoptosis was more pronounced in stattic combined with irradiation group than that in irradiation alone group. This result indicated that stattic enhanced the radiosensitivity probably through an apoptosis pathway. Therefore, the induction of apoptosis may be one of the mechanisms whereby stattic enhances the radiosensitivity of HCC cells.

## 5. Conclusion

In summary, this study suggested that stattic could reduce the expression of p-STAT3 and cell survival, enhance radiosensitivity, and inhibit radio-induced migration and invasion in HCC cells. Our results suggest that stattic is a potential radiosensitizer for the radiotherapy of HCC.

## Figures and Tables

**Table 1:** The radiosensitizing effect of stattic in HCC cells.

	Groups	*D*0 (Gy)	*Dq* (Gy)	SF_2_	SER_*D*0_
Hep G2	IR	2.25 ± 0.23	1.54 ± 0.13	0.66 ± 0.09	1.00 ± 0.12
	IR + stattic	1.44 ± 0.12	0.85 ± 0.11	0.40 ± 0.05	1.56 ± 0.16
BEL-7402	IR	4.07 ± 0.56	0.87 ± 0.12	0.68 ± 0.08	1.00 ± 0.15
	IR + stattic	2.55 ± 0.22	0.15 ± 0.02	0.47 ± 0.05	1.60 ± 0.18
SMMC-7721	IR	2.38 ± 0.25	1.95 ± 0.14	0.72 ± 0.08	1.00 ± 0.13
	IR + stattic	1.41 ± 0.16	0.82 ± 0.10	0.50 ± 0.04	1.69 ± 0.18

IR: irradiation, *D*0: lethal dose (Gy), *Dq*: quasi-threshold dose (Gy), SF_2_: survival fraction values at 2 Gy, and SER: sensitization enhancement ratio.
